# Protocol for a randomised, double-blind, placebo-controlled study of grass allergen immunotherapy tablet for seasonal allergic rhinitis: time course of nasal, cutaneous and immunological outcomes

**DOI:** 10.1186/s13601-015-0087-2

**Published:** 2015-12-17

**Authors:** Esther Helen Steveling, Mongkol Lao-Araya, Christopher Koulias, Guy Scadding, Aarif Eifan, Louisa K. James, Alina Dumitru, Martin Penagos, Moisés Calderón, Peter Sejer Andersen, Mohamed Shamji, Stephen R. Durham

**Affiliations:** Allergy and Clinical Immunology, Section Inflammation, Repair and Development, National Heart and Lung Institute (NHLI), Imperial College London, Dovehouse Street, London, SW3 6LY UK; Allergy and Clinical Immunology, Randall Division of Cell and Molecular Biophysics, King’s College London, New Hunt’s House, Guy’s Campus, London, SE1 1UL UK; ALK Abello, Bøge Alle 6, 2970 Hørsholm, Denmark

**Keywords:** Sublingual immunotherapy tablet, GRAZAX, Allergy, Hay fever, Allergic rhinitis, Randomised, Double-blind, Placebo, CTIMP, Phleum pratense

## Abstract

**Background:**

Seasonal Allergic Rhinitis is characterised by inflammation of the nasal mucosa upon exposure to common aeroallergens, affecting up to 20–25 % of the population. For those patients whose symptoms are not controlled by standard medical treatment, allergen specific immunotherapy is a therapeutic alternative. Although several studies have shown changes in immunologic responses as well as long term tolerance following treatment with a sublingual allergy immunotherapy tablet, a detailed time course of the early mechanistic changes of local and systemic T and B cell responses and the effects on B cell repertoire in the nasal mucosa have not been fully examined.

**Methods/design:**

This is a randomized, double-blind, single-centre, placebo controlled, two arm time course study based in the United Kingdom comparing sublingual allergy immunotherapy tablet (GRAZAX^®^, ALK-Abello Horsholm, Denmark) plus standard treatment with placebo plus standard treatment. Up to 50 moderate to severe grass pollen allergic participants will be enrolled to ensure randomisation of at least 44. Further, we shall enrol 20 non-atopic volunteers. Screening will be completed before eligible atopic participants are randomised to one of the two treatment arms in a 1 to 1 ratio. The primary endpoint will be the total nasal symptom score assessed over 60 min following grass pollen nasal allergen challenge after 12 months of treatment. Clinical assessments and/or mechanistic analyses on blood, nasal fluid, brushing and biopsies will be performed at baseline at 1, 2, 3, 4 (coinciding with the peak pollen season), 6 and 12 months of treatment. After 12 months of treatment, unblinding will take place. Those atopic participants receiving active treatment will continue therapy for another 12 months followed by a post treatment phase of 12 months. Assessments and collection of biologic samples from these participants will take place again at 24 and at 36 months from the start of treatment. The 20 healthy, non-atopic controls will undergo screening and one visit only coinciding with the 12 month visit for the atopic participants.

**Discussion:**

The trial will end in April 2017. The trial is registered with ClinicalTrials.gov and the trial identifying number is NCT02005627.

Trial registration: Primary Registry: ClinicalTrials.gov, Trial Identifying number: NCT02005627, Secondary identifying numbers: EudraCT number: 2013-003732-72 REC: 13/EM/0351, Imperial College London (Sponsor): 13IC0847, Protocol Version 6.0, Date: 16.05.2014

**Electronic supplementary material:**

The online version of this article (doi:10.1186/s13601-015-0087-2) contains supplementary material, which is available to authorized users.

## Background

Seasonal allergic rhinitis (SAR) is an IgE-mediated inflammatory disease characterised by itching, sneezing, nasal discharge and congestion. Early and late phase responses (EPR and LPR) occur upon exposure to common aeroallergens [1]. A substantial increase in the prevalence of SAR has been reported in industrialised countries, including Western Europe [1] and it is believed to affect up to 20–25 % of the population, with an estimated 80 million sufferers in Europe [1]. SAR has been shown to impact quality of life and impair learning performance in school children [2]. The current management of SAR consists of pharmacotherapy such as antihistamines and corticosteroids [3]. Also the avoidance of aeroallergens such as staying indoors may be beneficial. For those patients whose symptoms are not controlled by standard medical treatment, allergen specific immunotherapy (SIT) is a therapeutic alternative [4]. In recent years, the sublingual route has been shown to be effective and in the case of GRAZAX^®^ sublingual allergen specific immunotherapy (SLIT)-tablets to induce long-term remission [5]. Although adequately powered head to head studies have not been performed, the effects of subcutaneous allergen-specific immunotherapy (SCIT) and SLIT-tablet may be comparable, whereas SLIT-tablets are more convenient and have a better safety profile such that it may be administered in the patient’s home [6].

Changes in cellular as well as humoral responses play a role in the short term and long term efficacy of SIT. A shift in the ratio of T-helper 2 (Th2) and T-helper 1 (Th1) has been observed both peripherally [7], and in local target organs [8], preventing allergic response after SIT.

While some of the underlying mechanisms of SLIT-tablets have not been studied in comparable detail to SCIT and remain unclear it is also assumed that the underlying immunological mechanisms may differ due to the different allergen administration routes. The oral mucosa is considered a site of natural immune tolerance. Previous findings suggest an interaction exists between Langerhans cells, epithelial cells, monocytes and oral DCs capable of producing IL-10, TGF-beta and activins and priming Treg cells [9].

In atopic individuals increased concentrations of allergen-specific IgE in serum as well as target organs has been observed. Under SCIT, especially during the first few weeks of treatment, allergen-specific IgE increases even further, while there is a decrease during the seasonal peak [10]. On the other hand IgG subclasses, especially IgG_1_ and IgG_4_, increase. In competing with IgE they seem to prevent interaction with the allergen, possibly mediated via the FCgammaRIIB receptor. Serum from these individuals inhibits IgE facilitated allergen binding (FAB) resulting in decreased T cell proliferation and reduced cytokine production and inhibition of basophil histamine release [11–14].

Congruently in SLIT-tablets, increases in allergen-specific IgE may occur within weeks of start of treatment and again blunting of the IgE increase during the season similar to what has been shown in SCIT. Also levels of serum IgG_4_, even though lower compared to SCIT, and facilitated allergen presentation (FAP) inhibition was increased [5]. Effects on T-reg cells are inconsistent [15]. When using a simplified assay, allergen-IgE complexes bound to Fcepsilon RII on the surface of B cells were detected by flow cytometry (IgE-FAB) and serum inhibitory activity for IgE-FAB increased under SLIT-tablets [16]. Our group has shown previously that successful SIT has been associated with increases in protective IgG_4_ and IgA_2_ responses and induction of antigen-specific regulatory T-cells (T-regs) [8, 17]. Furthermore, reduced levels of effector cells (mast cells, eosinophils, CD4+ T-cells) are recruited to the nasal epithelium after grass pollen allergen challenge [8, 16]. In an IL-10 and TGF-beta dependent manner, T-regs have been shown to suppress antigen-driven proliferative T cell responses and Th2 cytokine release [7, 18]. Also decreased eosinophil recruitment has been reported [19]. Elevated levels of IL-10 in parallel with clinical responsiveness preceding IgG_4_ was demonstrated in a SCIT compared to a placebo group with an increase as early as 2 weeks [19].

Despite these observed changes in cellular and humoral responses, a detailed time-course of SLIT-tablets-induced immunological changes has not yet been performed.

Effects of SIT have been reflected in previous studies in changes of the nasal mediators such as the chemokines tryptase and eosinophilic cationic protein (ECP) [21]. In a cross sectional study SIT patients compared to untreated allergics had reduced levels of early phase tryptase and eotaxin [22]. Other mediators such as the cytokine IL-5 in nasal fluid were suppressed after SCIT [21]. We were able to show that in SIT patients a reduced nasal fluid concentration of IL-4, IL-9 and trends for reduced IL-13 were present [22]. While peripheral IL-10 has been studied in SCIT, local changes of IL-10 after IT are less clear. While previous studies by Pilette have found fewer IL-10 mRNA+ cells in the nasal mucosa of allergics [23], Benson et al. reported increased levels of IL-10 [24]. In our recent cross sectional study no clear response of IL-10 in nasal fluid after NAC was seen [22]. Thus the time course in the first months of SLIT-tablets remains unclear and we aim to clarify this by examining a detailed early time course of chemokines and cytokines locally in SLIT-tablets in this prospective study.

Recently the emergence of a novel innate immune cell family, type 2 innate lymphoid cells (ILC2), originally defined in murine models, has attracted interest. Morphologically similar to lymphocytes they lack T-cell, B-cell, natural killer cell or other cell lineage markers [25]. Our group showed that peripheral ILC2s might also play a role in seasonal allergic rhinitis. An elevation of ILC2s was seen during the grass pollen season. In participants who received SIT levels were comparable to non-atopic controls and correlated with reported seasonal symptom severity [26]. Again a time course in a prospective SLIT-tablet study has not been examined before.

Although several studies have shown changes in immunologic responses following treatment with a SLIT-tablet, a detailed time course of local and systemic T and B cell responses and interactions, has yet to be fully determined. Moreover, although long-term clinical tolerance is associated with persistent blocking antibody responses in the periphery, the effect of SLIT-tablet on B cell repertoires in the target organ has not been fully examined. Recent advances in cloning of antibody genes from single B cells [27] and of sequencing of entire TCR and antibody repertoires present in biological samples [28] allow for detailed analysis of such repertoires in response to disease and subsequent treatment. Thus a number of questions in relation to SLIT-tablet can be addressed; Is there a clonal relationship between the induced changes in IgE and the IgG repertoires during SLIT-tablet, does the local IgE repertoire contract following tolerance induction? Is there infiltration or local expansion of protective B cells following treatment? Is persistent clinical tolerance associated with a restricted repertoire of high affinity blocking antibodies? In order to address these questions we aim to establish a detailed time course of the early response in T and B-cell responses and we aim to characterise local and peripheral B cell repertoires in patients undergoing SLIT-tablet over the time course of 2 years under treatment and 1 year post treatment.

### Hypotheses

We hypothesize that the analysis of the time course of SLIT-tablet over the first 4 months and up to 12 months will show early induction of T regulatory cells (T-regs) followed by later down regulation of Th2 responses and the emergence of ‘protective’ IgG/IgA antibodies both locally and in peripheral blood. This will correlate with surrogate clinical responses to treatment reflected by a reduction of the early phase response (EPR) following nasal allergen challenge (NAC) after 6 months and after 12 months of treatment. We furthermore assume that the sequencing of T and B cell receptor repertoires during the course of SLIT-tablet will demonstrate the appearance of distinct populations of grass pollen specific T and B cell clones.

### Primary objectives

Our primary objectives are to understand the time course of early immunological changes as well as long-term changes in B cell repertoire under grass pollen AIT in SAR corresponding to clinical surrogate markers of AIT efficacy with a reduction of the EPR following NAC.

### Secondary objectives

We aim to assess the early time course of changes in different clinical surrogate markers for AIT efficacy including both early-phase (0–60 min) and late-phase (8 h) measurements following nasal and intradermal allergen challenge. We shall also record medication and symptom scores during the pollen season. We are furthermore interested in establishing a molecular sensitisation profile in untreated and treated allergic individuals. We will document the presence and severity of any local and systemic side effects of AIT treatment.

### Trial design

This is a randomized, double-blind, single-centre, placebo-controlled study comparing SLIT-tablet plus standard treatment versus sublingual placebo plus standard treatment. The allocation of the atopic participants will be 1:1. We assume superiority for the SLIT-tablet over placebo.

## Methods

### Study setting

**Fig. 1:**
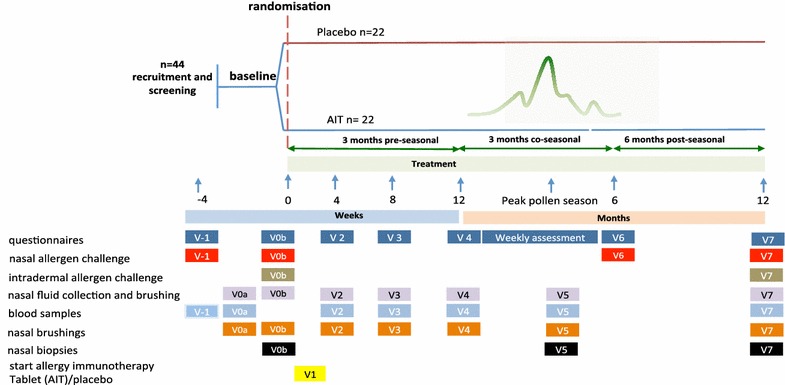
Study plan for atopic participants during the blinded phase of the treatment. *V* visit, *n* number

**Fig. 2:**
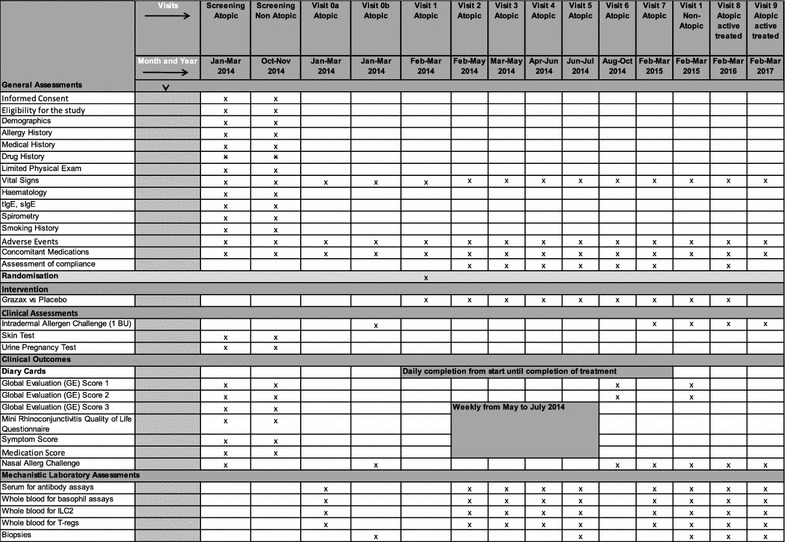
Study schedule and participant timeline. *GE* global evaluation score

The study will be conducted at the Royal Brompton Hospital (RBH), an academic hospital affiliated to the Imperial College London as sponsor (UK). We plan to enrol up to 50 participants in order to randomise at least 44 atopic participants. Additionally, we shall recruit 20 healthy, non-atopic volunteers. To assess a detailed time course of early immunological changes under SLIT-tablet clinical assessments and mechanistic analyses on blood, nasal fluid, brushing and biopsies will be performed at baseline at 1, 2, 3, 4 (coinciding with the peak pollen season), 6 and 12 months of treatment (Fig. 1). After 12 months of treatment, unblinding will take place. Those atopic participants receiving SLIT-tablet will continue therapy for another 12 months followed by a post treatment phase of 12 months. Assessments and sample collection from these participants will take place again at 24 and at 36 months from the start of treatment. The non-atopic volunteers will serve as a control for parameters collected at the 12 months’ time point. All participants will be provided with anti-allergic rescue medication throughout the pollen season 2014. Consent will be obtained in line with the Declaration of Helsinki. The Nottingham Ethics Committee has approved this study (reference 13/EM/0351). The duration of the trial is 3 years. The trial will end in March 2017 when the last subject undergoes the last study visit completing the open label follow up of 2 years after the 12 months blinded phase (Fig. 2).

### Eligibility criteria

Inclusion criteria of atopic participants:

1. Adults age 18–65 years.
2. A clinical history of grass pollen-induced allergic rhinoconjunctivitis for at least 2 years with peak symptoms in mid-May to mid-July.
3. A clinical history of moderate to severe rhinoconjunctivitis symptoms with or without mild seasonal asthma interfering with usual daily activities or with sleep.
4. A clinical history of rhinoconjunctivitis with or without mild seasonal asthma that remains troublesome despite treatment with either antihistamines or nasal corticosteroids during the grass pollen season.
5. Positive skin prick test response, defined as wheal diameter ≥3 mm, to timothy grass pollen.
6. Positive specific IgE, defined as IgE immunoCAP ≥0.7 ISU, against timothy grass pollen.
7. For women of childbearing age, a negative urine pregnancy test at the time of screening and willingness to use an effective form of contraception for the duration of involvement in the study.
8. The ability to give informed consent and comply with study procedures.
9. A positive grass pollen NAC test at screening as defined by a total nasal symptom score (TNSS) of at least 7/12 after 5 min with an allergen dose of 5000 BU/ml (in case at least 20 % of the screened individuals report a TNSS of ≤5 the cut-off will be lowered to a minimum of 5/12).

Inclusion criteria of non-atopic participants:

1. Adults age 18–65 years.
2. Negative skin-prick test response to timothy grass pollen and panel of aeroallergens.
3. Negative specific IgE, defined as IgE immunoCAP <0.35 ISU, against timothy grass pollen.
4. For women of childbearing age, a negative urine pregnancy test at the time of screening and willingness to use an effective form of contraception for the duration of involvement in the study.
5. The ability to give informed consent and comply with study procedures.

Exclusion criteria of atopic participants:

1. Previous grass pollen allergen immunotherapy.
2. Prebronchodilator FEV_1_ <70 % of predicted value at screening (out of grass-pollen season).
3. A clinical history of symptomatic allergic rhinitis and/or asthma caused by an allergen to which the participant is regularly and perennially exposed (e.g. cat dander).
4. Perennial asthma requiring regular inhaled corticosteroids.
5. Seasonal symptoms outside the grass-pollen season [e.g. hay fever during March–April suggestive of birch pollen allergy (during screening a panel of common aeroallergens such as house dust mite and birch pollen will be performed to allow exclusion of sensitisations to other aeroallergens of clinical relevance)].
6. History of emergency visit or hospital admission for asthma in the previous 12 months.
7. History of chronic obstructive pulmonary disease.
8. History of recurrent acute sinusitis.
9. History of chronic sinusitis.
10. At screening visit, current symptoms of, or treatment for, upper respiratory tract infection.
11. Current smokers or a history of ≥5 pack years.
12. History of life-threatening anaphylaxis or angioedema.
13. Ongoing systemic immunosuppressive treatment.
14. The use of any investigational drug within 30 days of the screening visit.
15. The presence of any medical condition that the investigator deems incompatible with participation in the study.
16. History of fish allergy with positive skin test and/or positive specific IgE test to vertebrate/finned fish (due to potential fish allergen exposure in SLIT-tablet).
17. Contraindications taking GRAZAX^®^.

Exclusion criteria of non-atopic participants:

1. Previous grass pollen allergen immunotherapy.
2. Prebronchodilator FEV1 <70 % of predicted value at screening (out of grass-pollen season).
3. A clinical history of symptomatic allergic rhinitis and/or asthma caused by an allergen to which the participant is regularly and perennially exposed (e.g. cat dander).
4. Perennial asthma requiring regular inhaled corticosteroids.
5. Seasonal symptoms outside or during the grass-pollen season.
6. History of emergency visit or hospital admission for asthma in the previous 12 months.
7. History of chronic obstructive pulmonary disease.
8. History of significant recurrent acute sinusitis.
9. History of chronic sinusitis.
10. At screening visit, current symptoms of, or treatment for, upper respiratory tract infection.
11. Current smokers or a history of ≥5 pack years.
12. History of life-threatening anaphylaxis or angioedema.
13. Ongoing systemic immunosuppressive treatment.
14. The use of any investigational drug within 30 days of the screening visit.
15. The presence of any medical condition that the investigator deems incompatible with participation in the study.

#### Intervention

SQ^®^ grass SLIT-tablet (GRAZAX^®^, ALK Abello Horsholm, Denmark) is a fast-dissolving tablet that is registered throughout Europe and North America for sublingual use in patients aged 5–65 years [20, 29, 30]. The tablet is administered sublingually and daily for a minimum of 2 months before and during the grass pollen season. In a double-blind trial of GRAZAX^®^ that included a 2 year follow up period efficacy was maintained for 2 post treatment years [29–32]. Even though SLIT-tablet has been shown to be safer than the injection route it is recommended that the first dose of GRAZAX^®^ is administered in the clinic. Subsequent doses are taken daily by the patient. Local side effects of itching and swelling in the mouth are common, occurring in up to 60 % of individuals within minutes and resolving within 1 h with a median half-life of approximately 10 days [29–31]. These side effects are, in general, well tolerated and require no treatment. In a recent large trial involving over 600 patients, the withdrawal rate was 5 % as compared to 3 % in placebo-treated patients [29, 30]. More severe local and systemic reactions have been reported, but they are excessively rare and no fatalities have occurred [33].

#### Formulation and packaging

GRAZAX^®^ is formulated as a freeze-dried oral lyophilisate disintegrating tablet for sublingual use. The active pharmaceutical ingredient is a standardized allergen extract derived from extraction and purification of grass pollen from timothy grass (*Phleum pratense*). The recommended dosage is one tablet containing 75,000 SQ-T^®^. The non-active ingredients consist of fish gelatin, mannitol, sodium hydroxide and water. The placebo is a tablet whose composition is identical to the GRAZAX^®^ tablet with the only exception being exclusion of the active pharmaceutical ingredient. GRAZAX^®^ and placebo tablets will be supplied in blister packs by ALK-Abello and packed into monthly dispensing packages containing 40 tablets. Two packages will be dispensed once every 2 months.

#### Dosing regimen

The first dose will be administered under an hour of observation in clinic followed by daily home administration. After unblinding, participants who received active treatment will receive SLIT-tablets for another 12 months. The participants who received placebo will be offered 24 months of SLIT-tablets.

#### Study medication accountability

The investigator is required to maintain adequate records of the disposition of the investigational product. An account of any drug accidentally or deliberately destroyed will be kept as a file note in the trial master file. Participants will be asked to return unused tablets at each study visit. The unused tablets will be counted by pharmacy. Participants will record their tablet intake on a daily diary.

#### Concomitant medications

During the pollen season the following medications will be provided: antihistamine (desloratidine 5 mg, up to once daily), nasal corticosteroid spray (fluticasone propionate aqueous nasal spray, 50 mcg per spray, up to 2 puffs twice daily) and ophthalmic antihistamine (olopatadine eye drops, 1 mg/ml, up to 1 drop per eye twice daily). We will assess the use of rescue medication by a weekly medication score. Oral corticosteroids (prednisolone 30 mg for 3–5 days), short-acting beta-agonists, inhaled corticosteroids and combination long-acting beta-agonists-steroids will only be provided to the participants after consultation with the investigators. Medication washout periods prior to NACs and biopsies need to be followed. During the study β-blockers, calcium channel blockers, tricyclic antidepressants, monoamine oxidase inhibitors or anti-IgE monoclonal antibody treatment are prohibited.

### Outcomes

Primary clinical endpoint

- The TNSS recorded over 60 min following grass pollen NAC.

Secondary clinical endpoints

- EPR of PNIF following NAC.
- LPR to intradermal allergen.
- EPR to intradermal allergen.
- Retrospective visual analogue scale (VAS) after the pollen season 2014.
- Retrospective global evaluation (GE) score after the pollen season 2014.

Exploratory clinical endpoints

- Combined weekly symptom and medication score over the course of the grass pollen season from May to July 2014.
- Mini Rhinoconjunctivitis Quality of life (mRQLQ) symptom score over the course of the pollen season 2014.
- Presence and severity of symptoms due to sublingual treatment.
- VAS over the course of the 2014 grass pollen season from May to July 2014.
- EPR following NAC at baseline, 6 months and 12 months.

Secondary mechanistic endpoints

- Grass allergen-induced basophil activation.
- Proportion of allergen-specific phenotypic Treg cells in peripheral blood.
- The inhibitory bioactivity of sera against allergen-IgE complex formation and binding to B-cell.
- The frequency of type 2 innate lymphoid cells.
- Concentration of serum total IgE and grass pollen specific IgG_4_ and IgE level.
- Cytokine concentration in peripheral blood.
- Concentration nasal fluid grass pollen specific IgG_4_ and IgE level.
- Cytokine RNA in nasal brushing.
- Single cell antibody cloning of local nasal B cells.
- T and B cell receptor sequencing.

### Sample size

Inclusion of 20 participants per group will give greater than 90 % power (p = 0.05) to detect a 40 % reduction in the nasal EPR after NAC (see Durham et al. [8]). Based on more recent studies (Scadding et al., unpublished data; mean 4.63, standard deviation 1.65 for area under the curve (AUC) 0-60 min post NAC in 14 allergic volunteers), inclusion of 13 patients per group will provide 80 % power to detect a 40 % reduction in AUC after NAC, whereas inclusion of 22 patients per group will provide 80 % power to detect a 30 % reduction. We will include 20 healthy, non-atopic controls to match the two atopic groups.

### Recruitment, screening and enrolment

Recruitment of the participants will be within the RBH, Imperial College London, and via local media. Potential atopic participants will be invited to visit the trial website (http://www.hayfeverstudy.com) for registration. They will be invited to attend the Research Unit at the RBH for a formal screening visit.

### Assignment of interventions

#### Allocation sequence generation

The randomisation list was produced by a statistician using Stata 10.1 without stratification or blocks and with an allocation of 1:1.

#### Allocation concealment mechanism

The sequence of the randomisation numbers is kept in sealed envelopes independent of the investigators.

#### Implementation

The packaging company will label the tablet packages according to the randomisation list. Single sealed envelopes containing the participants’ randomisation code are located at the RBH pharmacy.

#### Unblinding

Blinding of the investigators, outcome assessors, pharmacists, data analysts and trial participants will be maintained throughout the first 12 months of the study. Adverse events (AEs) that are considered serious, unexpected and at least possibly related to the medication would have to be unblinded. The study will be unblinded by the trial statistician after the final visit of the primary endpoint assessment at 12 months of treatment.

#### Withdrawal of participants/stopping rules and urgent safety measures

Participants have a right to withdraw at any time and may be withdrawn at the investigator’s discretion. Reasonable effort should be made to contact any participant lost to follow-up. The information collected before the withdrawal will be included in analysis. Participants will not be replaced. The PI will have the authority to deviate from the protocol if doing so relates to the immediate safety of a participant, where continuing to follow protocol would put that participant at risk. If any of the following criteria are met, study enrolment and study therapy will be suspended: death in any participant, where death is attributed in any way to study therapy or intervention; grade 4 anaphylaxis as defined by the World Allergy Organization (WAO) [34]. Study therapies will be discontinued for any of the following reasons: two or more occurrences of grade 3 or above systemic allergic reactions as defined by the WAO [34]; any AE that presents an unacceptable consequence to the participant; an illness that requires treatment not consistent with protocol requirements; inability to comply with the study protocol and pregnancy.

### Data collection, management and analysis

#### Data collection methods of the primary efficacy parameter

The primary efficacy parameter will be the EPR measured by the TNSS at 0, 5, 15, 30 and 60 min following grass pollen NAC in SLIT-tablet-, versus placebo-treated atopic participants at 12 months of treatment. The NAC will be performed with Aquagen SQ (ALK 225) Timothy grass pollen, *Phleum pratense*, ALK-Abelló freeze dried extract (Cat. no. 1001862, ALK-Abelló, Denmark). Fresh extracts will be reconstituted in albumin-based diluent (ALK-Abelló) on a twice weekly basis at a concentration of 100,000 SQ-U/30,000 BU per ml. On a daily basis, a fresh dilution of 5000 BU/ml in normal saline will be prepared and added to a nasal applicator device (Bidose, Aptar Pharma/Pfeiffer, Germany), (100 µl/pump application). A single spray will be applied to each nostril.

#### Data collection methods of the secondary clinical efficacy parameters

- PNIF measured at 0, 5, 15, 30 and 60 min (EPR) following grass pollen NAC at baseline and at 12 months.
- Mean LPR to intradermal testing recorded as the mean diameter of the swelling (longest diameter plus perpendicular diameter, exclude pseudopods) measured after 8 h of allergen challenge at baseline and after 12 months of treatment. Intradermal skin tests are performed by injecting 1 BU (0.02 ml of 50 BU/ml) of allergen into the skin of the outer surface of the forearms.
- Mean EPR to intradermal testing recorded after 15 min of allergen challenge at baseline and after 12 months of treatment.
- VAS after the pollen season 2014 (‘How has your hayfever been overall during the last pollen season?’); single measurement and delta (comparing baseline with after pollen season time point 2014).
- GE score (‘How has your hayfever been during the last pollen season compared to the previous season?’) after the pollen season 2014.

### Exploratory endpoints

The endpoints listed below will compare the following groups using the same statistical methodology as described for the primary endpoint: SLIT-tablet versus placebo.

- Symptoms and medication scores will be self-assessed weekly during the pollen season from May 2014 until end of July 2014 by the atopic participants. The symptom score will encompass nose and eye symptoms recorded on a scale from 0 to 3 (with a score of 0 indicating no symptoms, 1, 2 and 3 indicating mild, moderate and severe symptoms respectively). The maximum score is therefore 18. Participants will be asked to use the rescue medication received by us on an as required basis only, starting with the antihistamines (tablets and/or eye drops) and if this does not suffice adding treatment with nasal spray. In case this still does not relieve their symptoms they will be advised to contact the trial team to start prednisolone 30 mg 3–5 days. Medication use will be recorded in weekly questionnaires by participants and a medication score will be calculated: desloratadine, 5 mg, up to 1 tablet daily and/or olopatadine eye drops, 1.0 mg/ml, up to 1 drop per eye twice daily (1 point per day); fluticasone nasal spray, 50 mcg per spray, up to 2 sprays per nostril twice daily (2 points per day); and prednisone, 5 mg per tablet, up to 6 tablets per day (3 points per day). The maximum daily medication score will be 3. The maximum weekly score will be 21. 21 will be divided by 7 and multiplied by 6, which gives us a maximum score of 18, to allow a comparison between symptom and medication scores, since maximum scores for symptoms and medications are different in magnitude, as recommended by WAO guidelines. A combined symptom and medication score will be calculated by dividing both scores by 6, summarize of the results and divide by 2 [35]. Composite scores in each treatment group during the pollen season (beginning of May to end of July) and during the peak pollen season (approximately mid-June, defined as the max 14 day rolling average pollen count during the season) will be compared.
- MiniRQLQ scores will be collected weekly during the pollen season. Composite scores in each treatment group during the peak pollen season (beginning of May to end of July) will be compared. Furthermore the maximum RQLQ will be compared.
- Symptoms and severity of symptoms after intake of the daily tablets will be recorded on a daily diary cards. The type of symptom (itchy mouth, swelling of the mouth or other) and the WAO Grade of the local symptoms will be calculated daily [36]. The duration of the symptoms in days and the duration of symptoms in minutes will be specified.
- VAS of overall hay fever symptoms in the last week will be collected weekly during the pollen season. Composite scores in each treatment group during the peak pollen season (beginning of May to end of July) as well as the maximum will be assessed.
- The TNSS measured at 0, 5, 15, 30 and 60 min (EPR) following grass pollen NAC at baseline, at 6 and at 12 months will be assessed similar to the primary endpoint.

#### Secondary mechanistic parameters

Grass pollen specific immunological markers in serum, nasal fluid and nasal brushings will be evaluated at baseline, after 1, 2, 3 months, during the peak pollen season (at 4 months) and after 12 months of treatment. Blood will be collected via venepuncture during the study visit.

- Basophil activation will be assessed by expression of the surface markers CD107a, CD63 and CD203c on basophils (CRTH2+ CD3− CD303− cells) in the whole blood via flow cytometry.
- The inhibitory bioactivity of sera against allergen-IgE complex formation and binding to B-cell is evaluated by using IgE-facilitated allergen binding (FAB) assay.
- The frequency of type 2 innate lymphoid cells is measured by immunostaining with fluorochrome monoclonal antibodies and determined using multicolor flow cytometric analysis.
- Concentration of serum total IgE and grass pollen specific IgE and IgG_4_ level will be measured on a CAP FEIA system (Thermo-Fisher, Uppsala, Sweden).
- PBMCs are isolated and cultured for 6 days and cytokine concentrations specifically IL-10 will be measured in culture supernatants by means of ELISA.
- The collection and preparation of nasal fluid will be performed with a sterile synthetic polyurethane sponge (Zuschnitt Schaumstoff RG27 grau, Gummi-Welz GmbH & Co, Germany, ISO 5999, 1982 sponge), inserted into each nostril. Sponges will be left in place for 5 min before removal to allow absorption of nasal secretions, and then transferred to centrifuge tubes with indwelling 0.22 μm cellulose acetate filters (Costar Spin-X, Catalogue no. 8161, Corning, NY, USA). Tubes will be kept briefly on ice, then 75 µl of assay buffer (Millipore; Catalogue No. L-AB) will be added on top of the sponge into each tube before being centrifuged at 4500 RCF for 15 min at 4 °C. The volume of fluid collected will be calculated, aliquoted into Micrewtubes^®^ (cat no.: T336-2S, Simport, Beloeil, Canada) and stored at −80 °C. Concentration of nasal fluid grass pollen specific IgG_4_ and IgE level will be measured on a CAP FEIA system (Thermo-Fisher, Uppsala, Sweden). Nasal fluid analyses for cytokines and chemokines using a human cytokine/chemokine magnetic bead panel 96-well plate assay (Milliplex Map Kit; Millipore) and a Luminex xMAP Magpix platform (Millipore) will be performed.
- Biopsies will be performed at baseline, during the peak pollen season, after 12 months, 24 months of treatment and 12 months post treatment. Single cell antibody cloning will be performed according to standardized methods developed by the commercial suppliers (http://www.irepertoire.com or http://www.adaptivebiotech.com). Cloning of antibody heavy and light variable genes from single B cells will be done essentially as previously described [27]. Isotype switched B cells will be isolated from enzymatically dissociated nasal biopsy tissue by fluorescence activated cell sorting (FACS). Matched heavy and light chain transcripts will be amplified from individually sorted single B cells by RT-PCR. PCR products will be sequenced and selected antibodies will be cloned and produced by recombinant expression. Allergen specificity will be determined by ISAC microarray and antibody—allergen binding affinities will be determined by surface plasmon resonance. In parallel, RNA will be isolated from a second nasal biopsy and from PBMC and converted to cDNA. Heavy (IgA, IgG, IgM and IgE) chain variable region transcripts will be amplified using isotype-specific PCR and sequenced in parallel using high-throughput Illumina MiSeq sequencing.

The start of the pollen season will be defined retrospectively for the purpose of the analysis. Start of the pollen season is defined as the first 3 consecutive days with pollen count >10 grains/cm^3^, end of season is defined as the first consecutive days with pollen count <10 grains/cm^3^, start of peak pollen season is defined as the first 3 consecutive days with pollen count >30 grains/cm^3^, end of peak pollen season is defined as the first consecutive days with pollen count <30 grains/cm^3^.

### Data management

#### Statistical analysis

The statistical analysis plan will be finalised by the trial statistician prior to database lock. Primary analysis of treatment effect will be conducted under the intention-to-treat (ITT) principle defined as all randomized participants. The per-protocol (PP) sample will be defined as ITT sample participants in whom the primary endpoints were assessed. The Safety sample (SS) will be defined as all enrolled participants. The following groups will be compared: SLIT-tablet versus placebo; SLIT-tablet versus placebo versus non-atopic controls.

#### Analysis of the primary endpoint

This longitudinal data will be analysed using approaches that model the correlation structure such as models for the mean response and random coefficient models.

### Secondary and exploratory endpoints

All secondary analyses will be treated as supportive. P values will be presented for the secondary endpoints but will not be adjusted for multiplicity.

### Secondary mechanistic endpoints

Grass pollen specific immunological markers in serum, nasal fluid and nasal brushings and nasal biopsies at 12 months of treatment will be evaluated.

### Monitoring

#### Data monitoring

The data will be monitored regularly twice in the first year and once yearly in the years following the first year by Imperial College, the sponsor of this trial. Monitoring by the REC and MHRA may also occur.

#### Procedures for recording and reporting adverse events

AE will be recorded and severity and relation to study participation will be assessed. SAE data will be collected, faxed to the Sponsor and transcribed to eCRF. Please also refer to Additional file 1.

#### Adverse events that do not require reporting

These include the following:

- Seasonal symptoms such as itching, sneezing and wheezing.
- Not bothersome local symptoms after SLIT-tablet intake.
- Non-bothersome reactions to the non-invasive nasal secretion collection.
- Not bothersome allergic reactions after Intradermal skin tests.
- Bleeding responding to local finger pressure and discomfort responding to paracetamol after nasal biopsy.

#### Ethics and dissemination

The study has been approved by the Nottingham Ethics Committee (reference 13/EM/0351).

#### Amendments

Study amendments have been approved by the REC, the R and D Office and the MHRA where appropriate.

#### Consent

A participant information sheet will be provided to each person to read at least 24 h prior to the screening visit. Written IC will be obtained prior to any study specific procedures taking place.

#### Confidentiality

Data will be handled, computerised and stored in accordance with the Data Protection Act, 1998.

#### Access to data

Database access will be restricted through passwords to the authorised research team.

#### Data sign off

The PI will provide an electronic signature for each patient eCRF once all queries are resolved and immediately prior to database lock.

## Discussion

This protocol will allow us to investigate the time course of the first 4 months after start of SLIT-tablet. Further we will have the opportunity to look at B cell repertoire after 12, 24 and 36 months of treatment. The trial will end in April 2017.

## Additional file

10.1186/s13601-015-0087-2 Recording and reporting adverse events.

## Abbreviations

AE: adverse event
AIT: allergy Immunotherapy
AR: adverse reaction
eCRF: electronic case record form
EudraCT: European union drug regulating authorities clinical trials
EPR: early phase response
FAB: facilitated allergen binding
FEV1: forced expiratory volume in one second
GCP: good clinical practice
GE: global evaluation
ICH: International Conference on Harmonisation
IgE: immunoglobulin E
IMP: investigational medicinal product
ISU: ISAC standardized units
JRCO: Joint research compliance office
LPR: late phase response
MHRA: Medicines and healthcare products regulatory agency
NIHR: National institute for health research
NRES: National research ethics service
PEF: peak expiratory flow
Phl p: phleum pratense (Timothy grass)
PI: principal investigator
PNIF: peak nasal inspiratory flow
REC: Research ethics committee
SAE: serious adverse event
SAP: statistical analysis plan
SAR: seasonal allergic rhinitis
SAR: serious adverse reaction
SCIT: subcutaneous immunotherapy
SIT: specific immunotherapy
SLIT: sublingual immunotherapy
SOP: standard operating procedure
SSAR: suspected serious adverse reaction
SUSAR: suspected unexpected serious adverse reaction
TNSS: total nasal symptom score
URL: uniform resource locator
WAO: World Allergy Organization
