# Supplementary material for: Protocol for a randomised, double-blind, placebo-controlled study of grass allergen immunotherapy tablet for seasonal allergic rhinitis: time course of nasal, cutaneous and immunological outcomes
Source: Clin Transl Allergy. 2015 Dec 17;5:43. doi: 10.1186/s13601-015-0087-2 (PMC4682243; doi:10.1186/s13601-015-0087-2)
Supplement: Supplementary file 1 — 10.1186/s13601-015-0087-2 Recording and reporting adverse events. [file 13601_2015_87_MOESM1_ESM.docx]

## Additional file 1: Recording and Reporting Adverse events

ADDITIONAL INFORMATION:

Procedures for Recording and Reporting Adverse Events

The Medicines for Human Use (Clinical Trials) Regulations 2004 and Amended Regulations

2006 gives the following definitions:

Adverse Event (AE): Any untoward medical occurrence in a subject to whom a medicinal product has been administered including occurrences which are not necessarily caused by or related to that product.

Adverse Reaction (AR): Any untoward and unintended response in a subject to an investigational medicinal product which is related to any dose administered to that subject.

Unexpected Adverse Reaction (UAR): An adverse reaction the nature and severity of which is not consistent with the information about the medicinal product in question set out in:

The summary of product characteristics (SmPC) for that product (for products with a marketing authorisation) or the Investigator's Brochure (IB) relating to the trial in question (for any other investigational product).

Serious Adverse Event (SAE), Serious Adverse Reaction (SAR) or Unexpected Serious Adverse Reaction (USAR): Any adverse event, adverse reaction or unexpected adverse reaction, respectively, that results in:

- death
- Is life-threatening
- Required hospitalisation or prolongation of existing hospitalisation
- Results in persistent or significant disability or incapacity
- Consists of a congenital anomaly or birth defect

Although not a serious adverse event, any unplanned pregnancy should be reported via the

SAE reporting system as stated below.

Reporting Responsibilities

Imperial College London as the sponsor has delegated the delivery of the Sponsor’s responsibility for Pharmacovigilance (as defined in Regulation 5 of the Medicines for Human Use (Clinical Trials) Regulations 2004 to the Principal Investigator.

All SAEs, SARs and SUSARs (excepting those specified in this protocol as not requiring reporting) will be reported immediately by the Principal Investigator to the Sponsor, MHRA and REC in accordance with the current Pharmacovigilance Policy.

The Principal Investigator will report SUSARs to the sponsor, to the regulatory authorities (MHRA) and to the relevant ethics committee. Reporting timelines are as follows:

− SUSARs which are fatal or life-threatening must be reported not later than 5 days after the sponsor is first aware of the reaction. Any additional relevant information must be reported within a further 8 days.

− SUSARs that are not fatal or life-threatening must be reported within 15 days of the sponsor first becoming aware of the reaction.

The Principal Investigator (on behalf of the sponsor), will submit a Development Safety Update Report (DSUR) relating to this trial IMP, to the MHRA and REC annually.
